# Risk of Resource Failure and Toolkit Variation in Small-Scale Farmers and Herders

**DOI:** 10.1371/journal.pone.0040975

**Published:** 2012-07-26

**Authors:** Mark Collard, April Ruttle, Briggs Buchanan, Michael J. O’Brien

**Affiliations:** 1 Human Evolutionary Studies Program and Department of Archaeology, Simon Fraser University, Burnaby, British Columbia, Canada; 2 Department of Anthropology, University of Missouri, Columbia, Missouri, United States of America; New York State Museum, United States of America

## Abstract

Recent work suggests that global variation in toolkit structure among hunter-gatherers is driven by risk of resource failure such that as risk of resource failure increases, toolkits become more diverse and complex. Here we report a study in which we investigated whether the toolkits of small-scale farmers and herders are influenced by risk of resource failure in the same way. In the study, we applied simple linear and multiple regression analysis to data from 45 small-scale food-producing groups to test the risk hypothesis. Our results were not consistent with the hypothesis; none of the risk variables we examined had a significant impact on toolkit diversity or on toolkit complexity. It appears, therefore, that the drivers of toolkit structure differ between hunter-gatherers and small-scale food-producers.

## Introduction

Investigating the causes of toolkit variation is an important task for researchers interested in the evolutionary history and adaptive significance of human behavior. Variation in the number and intricacy of food-getting tools is one of the more obvious aspects of the ethnographic record [1], [2], and artifacts linked to the acquisition and processing of food dominate the archaeological record until the Holocene [3]. Thus, to understand both the ethnographic record and the archaeological record, we have to identify the causes of variation in subsistence technology.

Here we report a study of the possible causes of toolkit variation among small-scale farming and herding groups. Currently, little is known about this topic. A number of studies have examined the causes of cross-cultural variation in the number and intricacy of food-getting tools used by hunter-gatherers [1], [2], [4]–[12], but the causes of variation among the toolkits of farmers and herders have not been examined in any detail. Farmers were included in two previous toolkit-focused studies [2], [13], but one of them did not test any hypotheses regarding the causes of cross-cultural variation in toolkit structure [2], and the only tools examined in the other study were foraging implements [13]. Given that farmers and herders have outnumbered hunter-gatherers for several millennia and that food-production-related tools are therefore an important part of the Holocene archaeological record, the paucity of work on the causes of toolkit variation among farmers and herders is problematic.

We analyzed toolkit structure using the method that has been employed in most studies of hunter-gatherer toolkit variation [1], [2], [4]–[11]. Introduced by Oswalt in the early 1970s [1], [2], the method focuses on tools employed directly in the acquisition of food, which Oswalt termed *subsistants*. Oswalt divided subsistants into four categories: instruments, weapons, tended facilities, and untended facilities. Instruments are used to procure food that cannot run away or threaten its pursuer, such as plants or sessile animals. A digging stick is an example of an instrument. Weapons are designed to kill or maim potential prey that can escape or may harm its pursuer. Weapons include boomerangs, crossbows, and harpoons. Facilities are structures that control the movement of animals or protect them to a human’s advantage, such as a fish weir or a livestock pen. Tended facilities require continuous monitoring while in use (e.g., a fishhook), whereas untended facilities are capable of functioning without a human present and require only occasional monitoring (e.g., a deadfall trap). Oswalt created a further distinction between simple and complex subsistants. Simple subsistants do not change structurally during use, whereas complex subsistants have multiple parts that change position relative to one another during use.

Oswalt [1], [2] devised three measures of toolkit structure. The first is the total number of subsistants (STS), which is an indicator of the size, or what Torrence [6] and Shott [7] call the *diversity*, of a toolkit. The second is the total number of *technounits* (TTS). Formally, a technounit is an “integrated, physically distinct, and unique structural configuration that contributes to the form of a finished artifact” ([2], p. 38). More simply, technounits are the different kinds of parts of a tool. The total number of technounits included in a toolkit is a measure of its *complexity* [2], [6], [7]. Oswalt’s third measure of toolkit structure is the average number of technounits per subsistant (AVE). Again, this is a measure of toolkit complexity [2], [6], [7].

**Table 1:** Groups in sample.

Group	Country	Group	Country	Group	Country
Akamba	Kenya	Lur	Iran	Sema Naga	India
Aymara	Peru	Malay	Malaysia	Seminole	USA
Azande	Sudan	Malekula	Vanuatu	Sinhalese	Sri Lanka
Garo	India	Mapuche	Chile	Somali	Somalia
Gikuyu	Kenya	Mataco	Bolivia	Tanala	Madagascar
Guarani	Paraguay	Mam Maya	Guatemala	Tarahumara	Mexico
Gwembe Valley Tonga	Zambia	Monguor	China	Tikopia	Solomon Islands
Haddad	Chad	Ojibwa	Canada	Trukese	Micronesia
Hopi	USA	Okinawa	Japan	Tuareg	Algeria
Huron	Canada	Ovimbundu	Angola	Vietnamese	Vietnam
Jivaro	Ecuador	Pawnee	USA	Walapai	USA
Kapauku	Indonesia	Pima	USA	Yanomami	Venezuela
Kogi	Colombia	Pukapuka	Cook Islands	Yuma	USA
Korea	South Korea	Quichua	Ecuador	Zapotec	Mexico
Lepcha	India	Rwanda	Rwanda	Zuni	USA

Present-day country names are provided as a guide to the location of the groups.

**Figure 1:**
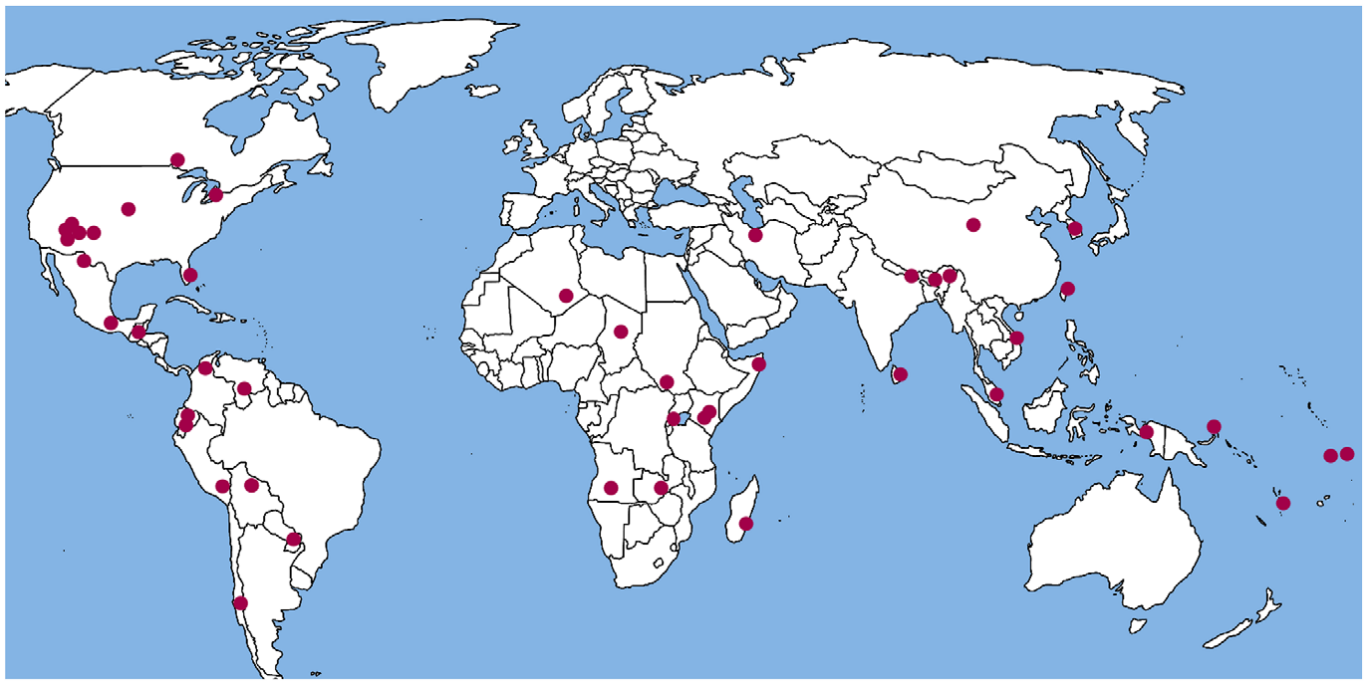
Distribution of the sample used in the study.

We focused on testing the hypothesis that risk of resource failure drives toolkit diversity and complexity. The risk hypothesis has its roots in Torrence’s “Time budgeting and hunter-gatherer technology” [4]. In this paper, Torrence hypothesized that as time stress increases, hunter-gatherers produce more specialized tools because they tend to be more effective. Because specialized tools generally have more parts than generalized tools, the production of more specialized tools increases not only toolkit diversity but also toolkit complexity. Subsequently, Torrence [5], [6] argued that time stress was likely only a proximate cause of toolkit variation and suggested that its ultimate causes are the timing and severity of risk of resource failure. Torrence argued further that the use of more specialized and therefore more elaborate tools reduces risk of resource failure. Thus, groups that experience high risk of resource failure will produce toolkits that are diverse and complex, whereas those that experience lower risk of resource failure will create simpler toolkits.

Several factors in addition to time stress and risk of resource failure have been hypothesized to influence the diversity and complexity of hunter-gatherer toolkits [1], [2], [4]–[12]. These include degree of reliance on mobile versus immobile resources [2], residential group mobility [7], [11], degree of reliance on terrestrial versus aquatic game [8], and population size [9], [12]. All of these hypotheses have received some empirical support [2], [7], [8], [11], [12]. However, when their explanatory power has been compared risk of resource failure has emerged as the major determinant of variation in hunter-gatherer toolkit diversity and complexity at the global scale [9], [11]. As such, testing the risk hypothesis is an obvious starting point for understanding the causes of toolkit variation among small-scale farmers and herders.

**Table 2:** Descriptive statistics and transformations.

Variable 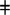	Mean	Std dev	*D*	*p*	Transformation	*D*	*p*
STS	44.93	18.18	.103	>.150	no	–	–
TTS	155.24	100.15	.183	<.010*	yes, square root	.120	.098
AVE	3.29	.76	.135	.040*	yes, square root	.112	>.150
HUNT	17.60	13.38	.110	>.150	no	–	–
FARM	23.09	14.80	.096	>.150	no	–	–
STORIRG	4.24	5.79	.169	<.010*	yes, square root	.088	>.150
LAT	20.25	13.54	.106	>.150	no	–	–
ELEV	853.40	857.08	.139	.036*	yes, square root	.069	>.150
CPB	18.07	9.70	.102	>.150	no	–	–
RAINAVG	97.68	87.35	.144	.028*	yes, Box-Cox^a^	.068	>.150
ET	16.84	3.39	.208	<.010*	yes, Box-Cox^b^	.096	>.150

The sample mean and standard deviation for each variable are presented. Kolmogorov-Smirnov normality tests were performed on each variable and the test statistic (*D*) and *p*-value reported. If the results of the Kolmogorov-Smirnov normality tests indicated a significant departure from normality, a transformation of the original data was performed and the results presented.

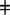
See text for an explanation of the variables.

* Indicates that the original data departed significantly from the expectations of a normal distribution based on the Kolmogorov-Smirnov normality test.

a A Box-Cox transformation with a λ of.337 (lower estimate.281, upper estimate.393) was used.

b A Box-Cox transformation with a λ of –2.022 (lower estimate –2.079, upper estimate –1.966) was used.

Extending the risk hypothesis to farmers and herders requires two assumptions to be made. One is that the principle that task-specific tools are more effective than multipurpose tools holds for food-producing tools as well as for tools used for hunting and gathering. The other is that farmers and herders experience similar levels of risk of resource failure as hunters and gatherers. Neither of these assumptions is particularly problematic. There is little experimental data on the relative effectiveness of task-specific versus multipurpose craft-produced tools, but there seems to be no reason why the principle should apply to hunting and gathering tools but not to food-producing tools. With regard to the risk experienced by food-producers, traditionally the transition to farming was conceptualized in terms of reducing food-related uncertainty and shortfalls [14], [15]. The ability of farmers to have some control over what and how much is planted, as well as when it is harvested, was argued to have reduced the rate of failure to meet dietary needs compared to hunting and gathering. However, in recent years it has become clear that farming is not less risky than hunting and gathering. For example, having reviewed human responses to environmental extremes and uncertainty, Low [16] concluded that hunter-gatherers are actually at *lower* risk of starvation and pathogen infection than are farmers. Similarly, Dirks [17] compared farmers and hunter-gatherers in terms of risk of resource failure and found that the levels of risk they experience are similar. Benyshek and Watson [18] carried out a comparable analysis to the one conducted by Dirks and reached similar conclusions. More recently still, Bowles [19] estimated the caloric costs and benefits of Neolithic cereal cultivation compared to hunting and gathering. His analyses indicated that early farming did not have a clear caloric benefit over hunting and gathering. Accordingly, there is reason to believe that small-scale food-producers experience similar levels of risk of resource failure to hunter-gatherers.

**Table 3:** Simple linear regression results for STS.

Variable	*r* ^2^	Slope (*β_1_*)	Standard error	Lower 95% CI for *β_1_*	Upper 95% CI for *β_1_*	*p*
LAT	.002	.057	.205	–.355	.470	.782
ELEV	.003	.070	.188	–.310	.449	.713
CPB	.003	–.097	.285	–.673	.478	.735
RAINAVG	.002	.436	1.694	–2.98	3.851	.798
ET	.002	–713.153	2259.596	–5270.062	3843.756	.754

The present study proceeded in a manner similar to those that have focused on the causes of toolkit variation among hunter-gatherers [4]–[9], [11]. We collected toolkit data for a global sample of ethnographically-documented small-scale farming and herding groups, and then collected data for several environmental variables that there is reason to believe influence the probability of resource failure. Subsequently, we regressed the toolkit variables on the risk variables and compared the resulting relationships with the main prediction of the risk hypothesis–that as risk of resource failure increases, toolkit diversity and complexity should increase.

**Table 4:** Multiple regression results for STS (overall model *r* ^2^ = .044; ANOVA results: *df* = 5,39, *F* = .355, *p* = .876).

Variable	Slope (*β_1_*)	Standard error	Lower 95% CI for *β_1_*	Upper 95% CI for *β_1_*	*p*
LAT	.354	.324	–.300	1.009	.280
ELEV	.259	.247	–.241	.758	.301
CPB	–.170	.322	–.821	.481	.601
RAINAVG	.863	1.96	–3.098	4.824	.662
ET	–3939.658	3921.616	–11871.876	3992.560	.321

**Table 5:** Simple linear regression results for HUNT.

Variable	*r* ^2^	Slope (*β_1_*)	Standard error	Lower 95% CI for *β_1_*	Upper 95% CI for *β_1_*	*p*
LAT	.001	–.023	.151	–.327	.281	.878
ELEV	.019	–.124	.137	–.401	.153	.372
CPB	.082	–.395	.201	–.801	.012	.057
RAINAVG	.004	.505	1.245	–2.005	3.015	.687
ET	.007	–889.560	1658.809	–4234.868	2455.748	.595

**Table 6 pone-0040975-t006:** Simple linear regression results for FARM.

Variable	*r* ^2^	Slope (*β_1_*)	Standard error	Lower 95% CI for *β_1_*	Upper 95% CI for *β_1_*	*p*
LAT	<.000	.011	.167	–.325	.347	.946
ELEV	.025	.158	.151	–.147	.463	.302
CPB	.059	.372	.226	–.083	.827	.107
RAINAVG	.003	.503	1.377	–2.274	3.280	.717
ET	<.000	–164.043	1840.727	–3876.223	3548.137	.929

**Table 7:** Simple linear regression results for STORIRG.

Variable	*r* ^2^	Slope (*β_1_*)	Standard error	Lower 95% CI for *β_1_*	Upper 95% CI for *β_1_*	*p*
LAT	.008	.007	.013	–.018	.033	.564
ELEV	.030	.014	.012	–.010	.037	.252
CPB	.031	–.021	.018	–.057	.015	.246
RAINAVG	.024	–.109	.106	–.322	.104	.309
ET	.005	64.285	142.496	–223.085	351.656	.654

**Table 8:** Multiple regression results for HUNT (overall model *r* ^2^ = .109; ANOVA results: *df* = 5,39, *F* = .958, *p* = .455).

Variable	Slope (*β_1_*)	Standard error	Lower 95% CI for *β_1_*	Upper 95% CI for *β_1_*	*p*
LAT	.034	.230	–.430	.499	.882
ELEV	–.073	.175	–.427	.281	.679
CPB	–.451	.229	–.913	.011	.056
RAINAVG	1.200	1.390	–1.612	4.012	.393
ET	460.897	2784.168	–5170.614	6092.409	.869

**Table 9 pone-0040975-t009:** Multiple regression results for FARM (overall model *r* ^2^ = .103; ANOVA results: *df* = 5,39, *F* = .893, *p* = .495).

Variable	Slope (*β_1_*)	Standard error	Lower 95% CI for *β_1_*	Upper 95% CI for *β_1_*	*p*
LAT	.177	.255	–.338	.693	.491
ELEV	.250	.195	–.144	.643	.207
CPB	.366	.254	–.147	.880	.157
RAINAVG	–.038	1.543	–3.159	3.084	.981
ET	–3318.802	3090.846	–9570.627	2933.024	.290

**Table 10:** Multiple regression results for STORIRG (overall model *r* ^2^ = .105; ANOVA results: *df* = 5,39, *F* = .914, *p* = .482).

Variable	Slope (*β_1_*)	Standard error	Lower 95% CI for *β_1_*	Upper 95% CI for *β_1_*	*p*
LAT	.022	.020	–.018	.062	.270
ELEV	.023	.015	–.008	.053	.143
CPB	–.025	.020	–.064	.015	.216
RAINAVG	–.036	.120	–.278	.206	.765
ET	–181.967	239.534	–666.470	302.536	.452

## Materials and Methods

The sample consisted of 45 groups, 12 from North America, 8 from South America, 10 from Asia, 10 from Africa, and 5 from Oceania (Table 1). The locations of the groups are shown in Figure 1. At the time the ethnographic data used in the study were collected, all the groups produced food primarily for subsistence rather than commercial sale and used craft-made rather than factory-produced tools.

We collected data on all foraging and food production-related tools used by the groups. This includes tools employed in irrigation, tools used to ward off birds and mammals from agricultural fields, tools used to process food for consumption, and tools used to prepare food for storage. For each group we calculated the total number of subsistants (STS), the total number of technounits (TTS), and the average number of technounits per tool (AVE). We also divided STS into the total number of tools used to obtain wild resources (HUNT), the total number of general farming tools (FARM), and the total number of tools used in food storage and irrigation (STORIRG). The main source of toolkit data was the digital version of the Human Relations Area Files (eHRAF), which is a Web-accessible, key word-searchable collection of ethnographies. Additional data were obtained from searches of hardcopy ethnographic sources not included in the eHRAF.

Next, we collected values for five risk variables: latitude (LAT), elevation (ELEV), average monthly rainfall (RAINAVG), effective temperature (ET), and the number of insect crop-pest species present in the groups’ countries, which we call “crop pest burden” (CPB). Other variables obviously could have been measured–for example, evapotranspiration rate or soil quality–but we considered these five to be an adequate ensemble of variables affecting probability of resource failure. Both the kinds of plants that can be grown and the yields of those plants are affected by latitude and elevation [20]. Rainfall, effective temperature, and the number of insect pests also affect farming yields [21]–[23]. Importantly, the variables include two of the risk variables that have been found to influence the diversity and complexity of hunter-gatherer toolkits (LAT and ET) [5], [6], [9], making it possible to directly compare our results with the previous work on the drivers of toolkit structure variation in hunter-gatherers.

Latitude and elevation data were collected from the same sources as the toolkit data. The values for average rainfall were obtained from several open-access sources of climatic information [24]–[28]. As far as possible, we used values for average rainfall from the same historical period as the toolkit data. Developed by Bailey [29], ET is a measure of relative warmth. It is calculated using the following equation:
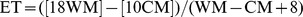
where WM is the mean temperature of the warmest month of the year, and CM is the mean temperature of the coldest month of the year. The first constant in the equation (18) is the minimum temperature for tropical climates for the coldest month of the year. The second (10) is the temperature limit of polar climates for the warmest month of the year. The third (8) is the minimum mean temperature at the beginning and end of the growing season. All the temperatures included in the equation for ET are in degrees Celsius. Values for the temperatures incorporated into effective temperature were obtained from the same sources as the values for average rainfall. Again, as far as possible, we used values for WM and CM from the same historical period as the toolkit data. The source of data for CPB was the Centre for Agricultural Bioscience International’s crop pest database [30], which contains country-level distribution data for approximately 900 insect crop-pest species. We selected a random sample of 100 species and counted the number of those species present in the countries occupied by the groups in the sample.

Subsequently, we ran both simple linear regression and multiple regression analyses to test the prediction that the diversity and complexity of toolkits used by small-scale farming and herding groups are positively related to risk proxies. These analyses used the toolkit variables (STS, TTS, and AVE) and three subsets of the number of subsistants (HUNT, FARM, and STORIRG) as the dependent variables, and the five risk variables (LAT, ELEV, RAINAVG, ET, CPB) as the predictors or independent variables. In the multiple regression analyses we used the enter model with all the risk proxies included as independent variables. LAT, ELEV, and CPB, were predicted to have a significant, positive impact on the toolkit variables, while RAINAVG and ET were predicted to have a significant, negative influence on the toolkit variables.

Prior to running the regression analyses we assessed the normality of the variables with the Kolmogorov-Smirnov test (Table 2). Six of the 11 variables departed significantly from the expectations of a normal distribution and therefore were transformed. We took the square root of four of them (TTS, AVE, STORIRG, ELEV) and used the Box-Cox transformation for the other two (RAINAVG, ET). The Box-Cox procedure estimates the best transformation to normality within the family of power transformations [31]. After transformation, the six variables had distributions that conformed to the expectations of a normal distribution according to the Kolmogorov-Smirnov test.

In the simple linear regression analyses we used an alpha correction method to reduce the possibility of committing type-II errors. We used Benjamini and Yekutieli’s [32] method of significance-level correction for multiple comparison tests. Narum [33] has shown that this method optimizes the reduction of both type-I and type-II error rates.

We conducted the Kolmogorov-Smirnov tests and Box-Cox transformations in Minitab 11. All regression analyses were run in PASW (SPSS) 18.

**Table 11:** Simple linear regression results for TTS.

Variable	*r* ^2^	Slope (*β_1_*)	Standard error	Lower 95% CI for *β_1_*	Upper 95% CI for *β_1_*	*p*
LAT	.001	.007	.038	–.070	.085	.847
ELEV	.002	.011	.035	–.060	.082	.762
CPB	.004	.023	.054	–.085	.131	.674
RAINAVG	.012	.226	.316	–.412	.864	.478
ET	.005	–194.744	423.560	–1048.932	659.444	.648

**Table 12:** Simple linear regression results for AVE.

Variable	*r* ^2^	Slope (*β_1_*)	Standard error	Lower 95%CI for *β_1_*	Upper 95% CI for *β_1_*	*p*
LAT	<.000	<.000	.002	–.005	.004	.900
ELEV	.001	<.000	.002	–.004	.005	.834
CPB	.093	.006	.003	<.000	.012	.042*
RAINAVG	.031	.022	.018	–.015	.059	.246
ET	.009	–15.409	24.749	–65.320	34.502	.537

* Significant at α = .05, but not significant when corrected for multiple unplanned comparisons using the Benjamini-Yekutieli method (α = 0.022).

**Table 13:** Multiple regression results for TTS (overall model *r* ^2^ = .050; ANOVA results: *df = *5,39, *F* = .414, *p = *.836).

Variable	Slope (*β_1_*)	Standard error	Lower 95% CI for *β_1_*	Upper 95% CI for *β_1_*	*p*
LAT	.064	.061	–.059	.186	.300
ELEV	.046	.046	–.047	.140	.322
CPB	.010	.060	–.112	.132	.871
RAINAVG	.220	.366	–.521	.960	.552
ET	–813.635	733.412	–2297.101	669.831	.274

**Table 14:** Multiple regression results for AVE (overall model *r* ^2^ = .133; ANOVA results: *df = *5,39, *F* = 1.194, *p = *.330).

Variable	Slope (*β_1_*)	Standard error	Lower 95% CI for *β_1_*	Upper 95% CI for *β_1_*	*p*
LAT	.002	.003	–.005	.009	.574
ELEV	.002	.003	–.003	.007	.506
CPB	.006	.003	–.001	.013	.070
RAINAVG	.009	.020	–.033	.050	.676
ET	–44.882	41.037	–127.886	38.122	.281

## Results

The prediction that the diversity of tools used by small-scale farming and herding groups should be positively related to risk proxies was not supported. The five simple linear regressions of the number of subsistants (STS) on the risk proxies (LAT, ELEV, CPB, RAINAVG, and ET) did not return any significant relationships (Table 3). Similarly, the multiple regression in which STS was the dependent variable and the risk proxies were the predictors indicated the overall model was not significant (*r* ^2^ = .044; ANOVA results: *df* = 5,39, *F* = .355, *p* = .876) and that none of the predictors had a significant impact on STS (Table 4).

The prediction that the diversity of tools used by small-scale farming and herding groups should be positively related to risk proxies was also not supported when the subsistants used for hunting (HUNT), general farming (FARM), and storage and irrigation (STORIRG) were analyzed separately. None of the simple linear regressions in which HUNT, FARM, and STORIRG were regressed on the risk proxies identified a significant relationship (Tables 5–7). Similarly, the three multiple regression analyses in which HUNT, FARM, and STORIRG were the dependent variables and the risk proxies were the predictors indicated the overall model was not significant and that none of the predictors was significantly related to the three subsets of subsistants (Tables 8–10).

Our analyses also did not support the prediction that the complexity of tools used by small-scale farming and herding groups should be positively related to risk proxies. The simple linear regressions in which the number of technounits (TTS) was regressed on the risk proxies did not identify any significant relationships (Table 11), nor did the simple linear regressions in which the average number of technounits per subsistant (AVE) was regressed on the risk proxies (Table 12). Results of the multiple regression analyses were consistent with those of the simple linear regression analyses. The multiple regression analysis in which TTS was the dependent variable and the risk proxies were the predictors indicated the overall model was not significant (*r* ^2^ = .050; ANOVA results: *df* = 5,39, *F* = .414, *p = *.836) and that none of the predictors was significantly related to TTS (Table 13). Similarly, the multiple regression in which AVE was the dependent variable and the risk proxies were the predictors indicated the overall model was not significant (overall model *r* ^2^ = .133; ANOVA results: *df = *5,39, *F* = 1.194, *p = *.330) and that none of the predictors was significantly related to AVE (Table 14).

## Discussion

The analyses reported here indicate that risk of resource failure does not have a significant impact on variation in either toolkit diversity or toolkit complexity among non-industrial farming and herding groups. They also indicate that risk of resource failure does not have a significant impact on variation in the diversity of hunting tools, general farming tools, or storage- and irrigation-related tools among such groups. These findings run counter to the risk of resource failure hypothesis.

The results of our analyses are strikingly different from the results of the global-scale analyses of variation in toolkit structure among hunter-gatherers that have been published to date. To reiterate, the latter collectively suggest that risk of resource failure is a major, if not *the* major, driver of toolkit diversity and complexity among hunter-gatherers [9], [11]. What accounts for this difference? Why should risk of resource failure seemingly drive variation in the toolkits of hunter-gatherers but not variation in the toolkits of food-producers?

One possibility is that the discrepancy is a consequence of the way in which we implemented our study. We think this is unlikely, however. The methods we used are the same ones used in the relevant hunter-gatherer studies. Similarly, there is sufficient overlap between the toolkit and risk variables we used and the ones employed in the hunter-gatherer studies that variable choice can be discounted as a potential explanation for the difference between our results and those of the hunter-gatherer studies. Most important, we included two risk proxies–latitude and effective temperature–that have been found to have a significant impact on hunter-gatherer toolkit diversity and complexity. The only other potential implementation-related cause of the discrepancy is the composition of our sample. If our sample were substantially smaller or more biased than the sample used in the hunter-gatherer studies, it might explain why ours does not support the risk hypothesis, whereas the hunter-gatherer studies support it. But such is not the case. The sample used in the hunter-gatherer studies comprises 20 groups from 4 regions (Africa, Australasia, Asia, and North America) [9], [11], whereas our sample consists of 45 groups from 5 regions (North America, South America, Asia, Africa, and Oceania). Thus, our sample is not only twice as large as the sample employed in the hunter-gatherer studies but also more geographically representative. It seems unlikely, therefore, that methodological differences account for the fact that our study did not support the risk hypothesis.

So far, we have identified two other potential explanations for the discrepancy between the results of our study and the results of the analyses of the drivers of global variation in hunter-gatherer toolkits. One is that food producers rely more heavily on non-technological practices to buffer themselves from risk of resource failure than hunter-gatherers do and that this affects the relationship between risk and toolkit structure. Among the non-technological practices we have in mind are spatial diversification, mixed farming, crop rotation, and intercropping. Spatial diversification–situating fields in several different locations instead of concentrating them in one area–allows a farmer to take advantage of microclimatic variations, thus reducing the risk of a total crop failure. Mixed farming, or using a combination of both cultigens and domestic animals, is another way for farmers to diversify and therefore reduce the likelihood of failing to meet their dietary needs. Crop rotation is the practice of growing different crops in the same field in different seasons, whereas intercropping is the practice of growing multiple crops in the same field. Crop rotation and intercropping have a number of outcomes that are beneficial in terms of risk reduction. Most notably, they protect against soil erosion, help maintain soil fertility, discourage crop pest infestation, and maximize land productivity [34].

Another potential explanation for the fact that the risk hypothesis is supported by the hunter-gatherer studies but not by ours is that farmers and herders experience higher levels of intergroup raiding and warfare than hunter-gatherers do and that this affects the relationship between toolkit structure and the environmental variables we used as risk proxies. On this hypothesis, intergroup raiding and warfare heighten the risk of resource failure for food-producing groups because food is likely to be stolen and economically active individuals are likely to be injured or killed, thereby reducing the number of people available to plant crops, build irrigation ditches, and so forth. The corollary of this is that the type of general environmental variables we used as risk proxies in our study can be expected to *underestimate* the level of risk faced by groups that experience high levels of intergroup raiding and warfare. This in turn means that the toolkit diversity and complexity values for these groups will be higher than expected given their latitude, effective temperature, and so on, and that the strength of the relationship between the toolkit variables and the environmental variables in the overall sample will be reduced.

In conclusion, the results of the study reported here are inconsistent with the hypothesis that risk of resource failure is the major determinant of variation in toolkit diversity and complexity in non-industrial societies. Thus there is a need to rethink the hypothesis in question. Either the hypothesis needs to be broadened to acknowledge that non-environmental factors such as intergroup raiding and warfare can impact risk of resource failure, or the hypothesis needs to be restricted to hunter-gatherer groups.
